# Letter to the editor: Need for a European network for enterovirus D68 surveillance after detections of EV-D68 of the new B3 lineage in Sweden and Italy, 2016 

**DOI:** 10.2807/1560-7917.ES.2017.22.2.30440

**Published:** 2017-01-12

**Authors:** Elena Pariani, Laura Pellegrinelli, Alessandra Di Cesare Merlone, Antonio Piralla, Fausto Baldanti, Sandro Binda

**Affiliations:** 1 Department of Biomedical Sciences for Health, University of Milan, Milan, Italy; 2 Unità Operativa Complessa di Pediatria, ASST Lariana Como, Italy; 3 Molecular Virology Unit, Microbiology and Virology Department, Fondazione IRCCS Policlinico San Matteo, Pavia, Italy

**Keywords:** EVD68, severe disease, acute flaccid myelitis, surveillance, B3 lineage, epidemiology

To the editor: In response to the rapid communication entitled ‘Outbreak of enterovirus D68 of the new B3 lineage in Stockholm, Sweden, August to September 2016’ by Dyrdak et al. [1] we would like to share our recent experience.

As the regional reference laboratory for acute flaccid paralysis (AFP) surveillance (Lombardy, northern Italy), a case of acute flaccid myelitis (AFM) came to our attention at the end of July 2016 when a previously healthy 4-year-old child with febrile (body temperature > 38 °C) respiratory illness and headache was hospitalised at a hospital in our region. The child’s condition suddenly worsened with the occurrence of severe neurological manifestations such as stiff neck, flaccid limb weakness associated with hyporeflexia, and bulbar muscle weakness, requiring intensive care unit care.

Clinical specimens (cerebral-spinal fluid, blood, nasal swab, nasopharyngeal aspirate, and stool) were collected at the onset of symptoms and laboratory tests ruled out poliomyelitis as differential diagnosis. Nasal swab, nasopharyngeal aspirate and stool samples all tested positive for enterovirus (EV) by real-time reverse transcription-polymerase chain reaction (RT-PCR) [2], and were then assessed for EV-D68 by a specific assay [3]. EV-D68 RNA was detected in all clinical samples examined and this result was further confirmed by sequence analysis of viral protein (VP)3/VP1/2A gene [4]. The phylogenetic analysis of the obtained sequence revealed the presence of an EV-D68 belonging to the recently described lineage B3 [5,6]. The nt sequence also shared high similarity (> 98.6% identity) with EV-D68 lineage B3 sequences.

This case evidences the occurrence of EV-D68 belonging to the new B3 lineage in Italy in the same period than the Swedish outbreak, that is during 2016 summer, and with similar neurological outcome as some cases reported by Dyrdak et al. [1]. Two years after the unexpected spread of EV-D68 worldwide in 2014, a number of epidemics occurred throughout Europe (Sweden, the Netherlands, and the United Kingdom) [7]. Since these epidemics were only reported by countries with an active EV surveillance the impact of EV/EV-D68 in Europe is likely to be more important. For other countries, such as Italy, this information is missing. The extent of circulation of EV-D68 is of course underestimated because of the scarcity of laboratories equipped to detect the virus and because of the poor awareness among healthcare workers of the problems the virus can cause.

It is now imperative to implement a surveillance system to monitor the spread and circulation of EV-D68 (in Italy and in Europe) due to its public health impact and consequences on childrens’ health. Such a surveillance scheme could be included in the framework of the AFP surveillance system [8], thus taking advantage of an existing and efficient network that includes skilled healthcare specialists, and that is endowed with equipped laboratories, with a wide expertise on EV detection and sequencing. It is becoming more and more essential to monitor not merely the virus circulation but also its molecular characteristics in order to identify any association between a specific lineage and disease severity.

The implementation of EV-D68 surveillance in the framework of the existing AFP network will entail: (i) a reevaluation of the case definition [8] to comprise severe respiratory illness and neurological impairment; (ii) a reappraisal of the panel of specimens to be collected (respiratory samples to be included always); (iii) the inclusion of EV-D68 identification assay(s) and sequencing, which should be within the laboratory capacity.

The realisation of a European network for EV-D68 surveillance will help to obtain the information needed to clarify viral epidemiology, to put in force proper control and preventive measures, so as to protect children and keep them safe and healthy.
